# Effects of Extracellular Self- and Nonself-DNA on the Freshwater Microalga *Chlamydomonas reinhardtii* and on the Marine Microalga *Nannochloropsis gaditana*

**DOI:** 10.3390/plants11111436

**Published:** 2022-05-27

**Authors:** Emanuela Palomba, Pasquale Chiaiese, Pasquale Termolino, Rosa Paparo, Edgardo Filippone, Stefano Mazzoleni, Maria Luisa Chiusano

**Affiliations:** 1 Department of Research Infrastructures for Marine Biological Resources, Stazione Zoologica “Anton Dohrn”, 80121 Naples, Italy; emanuela.palomba@szn.it; 2 Department of Agricultural Sciences, Università degli Studi di Napoli Federico II, 80055 Portici, Italy; chiaiese@unina.it (P.C.); filippon@unina.it (E.F.); stefano.mazzoleni@unina.it (S.M.); 3 Institute of Biosciences and Bioresources, National Research Council, 80055 Portici, Italy; pasquale.termolino@ibbr.cnr.it (P.T.); rosa.paparo@ibbr.cnr.it (R.P.)

**Keywords:** extracellular aggregates, extracellular DNA, microalgae stress, palmelloids, self-DNA inhibition

## Abstract

The role of extracellular DNA (exDNA) in soil and aquatic environments was mainly discussed in terms of source of mineral nutrients and of genetic material for horizontal gene transfer. Recently, the self-exDNA (conspecific) has been shown to have an inhibitory effect on the growth of that organism, while the same was not evident for nonself-exDNA (non conspecific). The inhibitory effect of self-exDNA was proposed as a universal phenomenon, although evidence is mainly reported for terrestrial species. The current study showed the inhibitory effect of self-exDNA also on photosynthetic aquatic microorganisms. We showed that self-exDNA inhibits the growth of the microalgae *Chlamydomonas reinhardtii* and *Nannochloropsis gaditana*, a freshwater and a marine species, respectively. In addition, the study also revealed the phenotypic effects post self-exDNA treatments. Indeed, Chlamydomonas showed the formation of peculiar heteromorphic aggregates of palmelloid cells embedded in an extracellular matrix, favored by the presence of DNA in the environment, that is not revealed after exposure to nonself-exDNA. The differential effect of self and nonself-exDNA on both microalgae, accompanied by the inhibitory growth effect of self-exDNA are the first pieces of evidence provided for species from aquatic environments.

## 1. Introduction

### 1.1. The Environmental Extracellular DNA

The extracellular DNA (exDNA) is “the DNA located outside the cell and originating from intracellular DNA by active or passive extrusion mechanisms or by cell lysis” [1]. It can be located in different contexts, like varied natural habitats (e.g., soil, sediments, oceans, and freshwater), independently from the originating cells, or still associated with them (like in extracellular matrices or in biofilms), and/or remain in the body of multicellular organisms [2]. Of note, it can be part of well-defined structures, for example, matrices of organic compounds produced by the native organism (e.g., neutrophil extracellular traps (NETs), root extracellular traps (RETs) and biofilms) [3,4,5], or even heterologous organic material (e.g., bound to soil humic acids [6]), or dissolved organic matters (DOM) found in marine ecosystems [7] or enclosed in organic organelles, such as native extracellular vesicles [8,9] or presumably heterologous ones.

The role of exDNA has been deeply investigated in soil, where it may originate from microorganisms (e.g., archaea, bacteria, photosynthetic microorganisms, fungi, protozoa, and soil invertebrates), and local or transient fauna as well as from plants [10]. In this context, it functions as a nutrient source especially in environments with low nutrient input [10] or serves as building blocks for the synthesis of new molecules, once cleaved by DNases [11]. In multicellular organisms, the exDNA can be of exogenous (dead or alive bacteria or viruses) as well as of endogenous origin [12], from lysed, apoptotic, or necrotic cells, or be actively released from living cells, as clearly demonstrating in highly proliferating cells [13] of plants [14,15], animals [16,17], and vertebrates of different phyla [18,19,20]. It can be free in the bloodstream [2,13], bound to cytoplasm constituents (e.g., lipids and proteins and other nucleic acid molecules, such as RNAs) [21], or to extracellular vesicles [13,17,22,23] but also physically attached to the outer leaflet of the plasma membrane [24,25]. Moreover, exDNA is also an important component of specific structures. Plant structures known as root extracellular traps (RETs) [4,15,26,27] have DNA among the different components (i.e., sugars, proteins, amino acids). These structures are actively produced by root cap cells and released in the external part of the root apex [28], appearing to have defensive roles [4,29]. Notably, similarly to RETs in plants, DNA has been found as component of defensive structures in animals, known as neutrophil extracellular traps (NETs) that consist of aggregates secreted by neutrophils composed by actin, histones, peroxidases, other proteins and DNA [3,30,31].

The exDNA is also a major component of the extracellular polymeric substance of both terrestrial and marine biofilms [32,33,34], as well as of biofilms of clinically relevant microorganisms [35]. ExDNA in biofilms can derive from lysed cells [36,37,38,39,40], or be actively released by microorganisms producing the biofilm itself [41,42,43,44,45,46]. In these contexts, it is a structural component required for proper biofilm formation and development [47] but it also has a protective role. Indeed, being negatively charged, it chelates cationic antimicrobials [48] and protects against aminoglycosides [49].

Beyond the well-known role as a nutrient and energy source, it has been highlighted by several findings that the exDNA can also function as a signalling molecule. For instance, in plants the presence of exDNA in the growth medium has been shown to enhance the growth of lateral roots and root hairs, thus controlling root physiology [50], while in animals, the exDNA acting as an endocrine molecule is able to modify the biology of the recipient cells (e.g., transforming normal cells into cancer cells) [51,52].

Furthermore, in multicellular organisms, the exDNA deriving from exogenous sources has been discussed in the context of pathogen-associated molecular patterns (PAMP), i.e., molecules originating from pathogens that are recognized by and activate cells of the innate immune system in both plants and animals. Differently, the exDNA of endogenous origin has been proposed to act as a “Damage-associated molecular pattern” (DAMP) [53,54,55] that, when present in an anomalous compartment, may trigger the activation of a non-infectious inflammatory response [53,54]. In animals, for example, the endogenous exDNA of nuclear or mitochondrial origin correlates with and/or determines various types of diseases [56,57,58,59], such as cancers [56] hypertension [59], and autoimmune diseases; among these are rheumatoid arthritis [60] and systemic lupus erythematosus [61].

### 1.2. The exDNA: A Focus on Its Location and Roles in Marine and Aquatic Ecosystems

ExDNA present in aquatic systems can be released via cell lyses or by active release [62,63,64]. In the marine context, the DNA not enclosed in living cells represents the 90% of the total DNA pool [65], and includes DNA released in situ (autochthonous) by sediment microbial communities or by local living species, as well as DNA of pelagic and of terrestrial origin transiently deposited to the seafloor (allochthonous) [66]. It can been found as free molecules in the water column, or in sediments, when complexed with colloids and particles [64,65,67,68,69], or in extracellular polymeric matrices of marine bacteria biofilms [70]. ExDNA preservation, concentration, and size depend on the physio-chemical features of the specific environment [63,64,67,68,69,71]. In general, its stability is influenced by the adsorption to particles [72,73], the presence of nucleases, mainly of bacterial origin [74,75], pollutants of anthropogenic origin [76], and by the temperature and the salinity of the water [77,78], since increased temperature and hypersaline environments enhances and limits exDNA degradation, respectively. Moreover, the exDNA can persist longer if it is physically bound to particles such as clay minerals and sand [79,80].

In aquatic contexts, exDNA is an important phosphate [69] and carbon [81] source for many marine microbial communities [69,82] and an important pool for genetic recombination and genetic modification via horizontal gene transfer [79,83]. It is part of extracellular matrices of marine biofilms [34] and can bear, for example, antimicrobial resistance genes whose exchange, mainly as plasmid DNA, may contribute to evolution and speciation of mainly prokaryotic organisms [79,83].

### 1.3. The Inhibitory Effect of Self-exDNA: A Novel Research Line

Recent investigations suggested new roles and effects of exDNA when independently considering conspecifics (self-exDNA) or non-conspecific (nonself-exDNA) molecules. In 2015, Mazzoleni and his colleagues demonstrated for the first time that the exposure to fragmented self-exDNA triggers inhibitory effects on conspecifics based on experiments in plants [84], while nonself-exDNA was demonstrated not to exert the same effect. In particular, within the same work, Mazzoleni and his colleagues addressed the question of the effect of DNA from phylogenetically related species. Indeed, they showed that *A. thaliana* exDNA inhibited the growth of *Lepidium sativum* seedlings and vice versa, but DNA did not inhibit *Acanthus mollis* growth [84]. Interestingly, *A. thaliana* and Lepidium belong to the same order (Brassicaceae), whereas the Acanthaceae belongs to a different order, the Lamiales. Similarly, it was also shown that *Capsicum chinense* DNA inhibited *Lactuca sativa* (both Asterales), whereas DNA extracted from *Acaciella angustissma* (Fabales) did not [85], and DNA from lima bean inhibited common bean growth whereas DNA from *Acacia farnesiana* did not [86]. This evidence supported the observation that the closer the organism phylogenetic distance, the higher the inhibitory effect of nonself-exDNA on the treated plant [84].

The inhibitory role of self-exDNA was also demonstrated in species of different taxonomic groups (such as bacteria, fungi, and invertebrates), suggesting that this is a general biological phenomenon [86]. Moreover, fragmented self-exDNA induces intracellular calcium signalling and plasma membrane depolarization [87], H_2_O_2_ production, and MAPK activation [55] in plants. Recently, it has been shown that after treatment with self-exDNA, tomato leaves activate a typical response to biotic stress characterized by an early response consisting of the plasma membrane depolarization, the increase of intracellular concentration of Ca^2+^ and K^+^ ions, and ROS production, accompanied by the modulation of genes involved in plant late responses to biotic stress [88].

Interestingly, Chiusano et al., in 2021, demonstrated that plants are able to distinguish self- from nonself-exDNA and, upon recognition, they trigger since the earliest stages after exposure produce different molecular responses [89]. Indeed, nonself-exDNA, after entering root cells, activates a hypersensitive response putatively evolving into systemic acquired resistance, while self-exDNA remaining outside root cells activates the chloroplast genome and ROS production eventually involving cell cycle arrest [89].

The suggested general phenomena causing different effects of self- versus nonself-exDNA exposure on living systems and the inhibitory effect of self-exDNA on conspecifics [84,89], that has been up to now mainly demonstrated for terrestrial species, although also confirmed in the green alga *Scenedesmus obliquus,* were here deeper evaluated in other aquatic species. Using *Nannochloropsis gaditana* and *Chlamydomonas reinhardtii* microalgae, both from aquatic environments and one from a marine and the other from a freshwater context, respectively, we here reported on the effects on cell growth of the exposure to self-exDNA and nonself-exDNA. Our experiments confirmed the differential response to self- and nonself-exDNA and highlighted novel phenotypic effects determined by cell treatments with self-exDNA.

## 2. Results

### 2.1. Self-exDNA Inhibits Growth of Freshwater and Marine Algae

**Figure 1:**
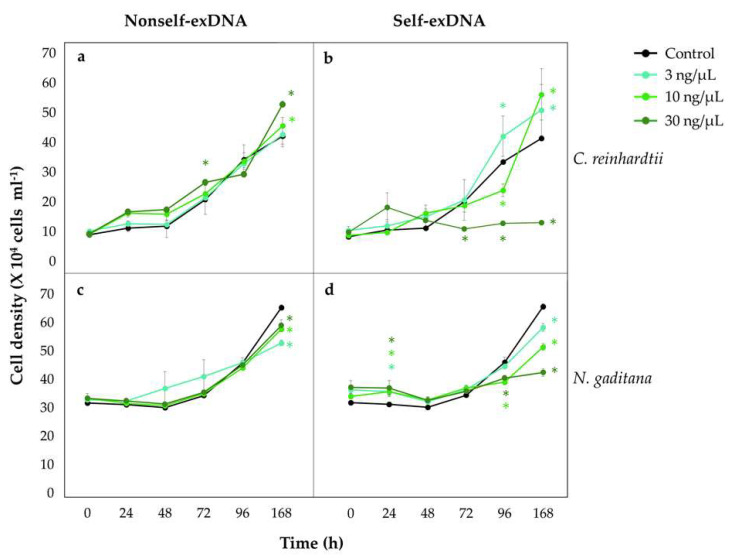
Cell density of *Chlamydomonas reinhardtii* (**a**,**b**) and *Nannochloropsis gaditana* (**c**,**d**) after the exposure to different concentrations of nonself- and self-exDNA. Controls (black lines and dots) are represented by microalgae grown in Bold’s Basal Medium (BBM) and Artificial Seawater Medium (ASW) for *C. reinhardtii* (**a**,**b**) and *N. gaditana* (**c**,**d**), respectively. Different coloured * indicate statistically significant differences between treatments and the control at each time (post hoc ANOVA, Dunnet test with *p* < 0.05).

*C. reinhardtii* growth curves (Figure 1a,b) showed that the cell density is not at affected at concentrations of nonself-exDNA (Figure 1a), as well as at lower concentrations (3 and 10 ng/µL) of self-exDNA (Figure 1b), following over time the same sigmoidal trends of the control. Conversely, the treatment with self-exDNA at 30 ng/µL significantly affected the culture cell density along the time course. In particular, differences between the self-exDNA and the control appeared more evident, with a decrease in cell density in treatments at 30 ng/µL that remained significantly lower than the control, and almost stable until 168 hpt, and at both 10 ng/µL at 96 hpt. It is worth noting that the higher cell density was in treatments at 3 ng/µL at 96 and 168 hpt, and in treatments at 10 ng/µL at 168 hpt.

At higher concentrations of nonself-exDNA (10 and 30 ng/µL), there were not significant differences compared with the control (Figure 1a), except at 168 hpt for the treatment at 30 ng/µL.

In *N. gaditana*, the treatments with nonself-exDNA at 3, 10, and 30 ng/µL slightly but significantly reduced cell density (Figure 1c), while the treatments with self-exDNA strongly affected the growth in a concentration-dependent manner, showing the highest growth inhibition at 30 ng/µL at 168 hpt (Figure 1d).

The evaluation of the growth rate inhibition versus the control (GRI (%)) at 168 hpt for *C. reinhardtii* and *N. gaditana* (Table 1) showed that, for both microalgae, the treatment with self-exDNA at 30 ng/µL strongly confirmed the growth inhibition (Table 1). The generation time (Tg) of *C. reinhardtii* at 168 hpt was around 3–4 days in all treatments and only in the treatment with self-exDNA at 30 ng/µL did it delay reaching the value of 22 days if compared with the control (Table 1). In *N. gaditana,* after all nonself-exDNA treatments, the Tg slightly elongated when compared with the control. Differently, after the treatment with self-exDNA, the Tg increased in a concentration-dependent manner, reaching a value at least six times higher than the control in self-exDNA at 30 ng/µL (Table 1).

### 2.2. Morphological Changes after Treatment with exDNA

**Figure 2:**
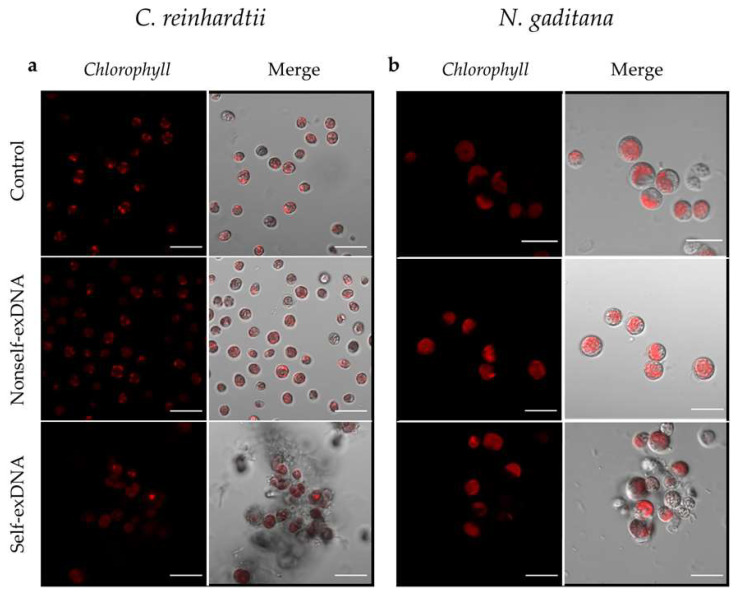
Microscopy analysis of *C. reinhardtii* (**a**) and *N. gaditana* (**b**) samples in controls and after treatments with nonself- and self-exDNA at 30 ng/µL. In red, the chlorophyll autofluorescence is shown (Ex/Em ^1^: 405/300–800); the composite is also shown (Merge). Microscope: Zeiss LSM 700, Oberkochen, Germany Excitation at 405, detection in the range 300–800, Objective: 63× 1.40 Oil Dic. ^1^ Ex/Em = Excitation/Emission.

**Figure 3:**
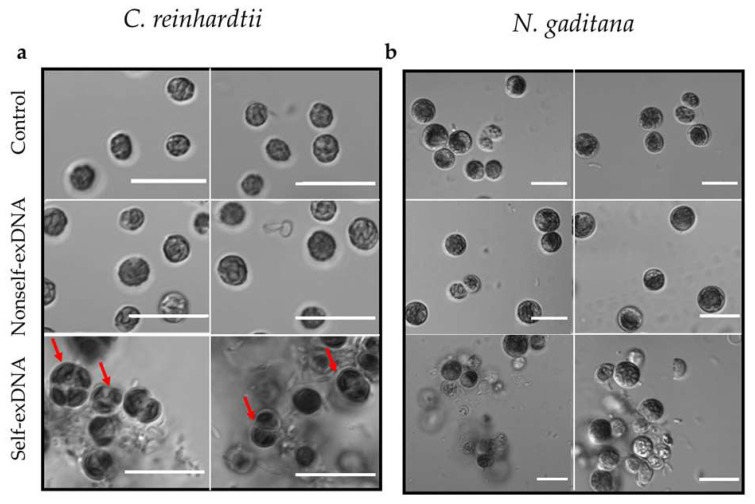
Light microscopy analysis of microalgae cells. *C. reinhardtii* (**a**) and *N. gaditana* (**b**) samples in controls and after treatments with nonself- and self-exDNA. Focus on the cell morphology showing aggregates and/or structural changes occurring only after treatments with self-exDNA at 30 ng/µL. In (**a**), red arrows indicate cells resembling a palmelloid stage (Scale bar = 10 µm). Microscope: Zeiss LSM 700, Oberkochen, Germany, Objective: 63× 1.40 Oil Dic.

*C. reinhardtii* cells treated at the three different concentrations of nonself-exDNA showed a morphology and a chlorophyll autofluorescence similar to the control (Figure 2a,b and Supplementary Figure S1a). This also appeared for the self-exDNA treatments at 3 and 10 ng/µL (Supplementary Figure S1a), while at 30 ng/µL the cells showed an altered morphology, appearing in a similar palmelloid phenotype and forming aggregates (Figure 2a,b and Figure 3a).

In *N. gaditana,* cells treated with the three different concentrations of nonself-exDNA showed a morphology and a chlorophyll autofluorescence similar to the control. At lower concentrations of self-exDNA treatments (3 and 10 ng/µL), the cells appeared with an altered organization (Supplementary Figure S1b) that became remarkable at 30 ng/µL, a concentration in which the formation of aggregates was evident too (Figure 2b and Figure 3b and Supplementary Figure S1b). In both species, the aggregates appeared as heteromorphous organizations with cells surrounded by a dense substance that appears similar to an extracellular matrix.

### 2.3. Microscopic Analysis of Aggregates after Self-exDNA Treatments

In order to investigate the nature of cellular aggregates and extracellular matrices observed after the treatment with self-exDNA at 30 ng/µL, we stained the aggregates formed in both species with DAPI (Supplementary Figure S3). The dye remarked the localization of DNA in the cell nucleus and in the organelles (plastids and mitochondria) in both control cells, and cells treated with both self- and nonself-exDNA. Moreover, in the self-exDNA treatments, the dye also stained all heteromorphous aggregates including the extracellular matrices, which were instead absent in nonself-exDNA treatments and in the control.

**Figure 4:**
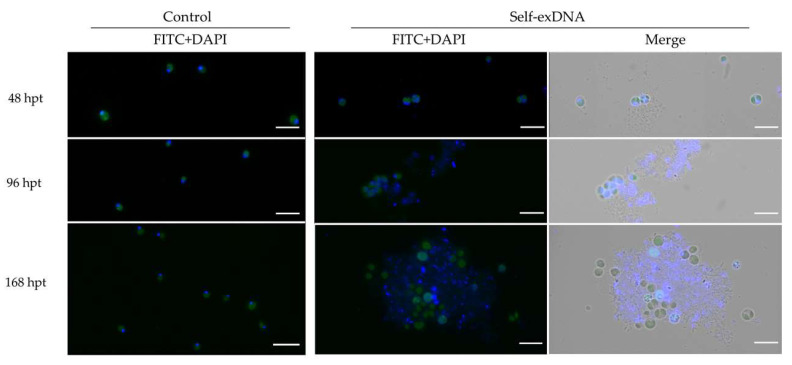
Fluorescence analysis of *C*. *reinhardtii* cells after treatment with self-exDNA at 30 ng/µL: focus on cellular aggregates formation. Cellular aggregates were analyzed using DAPI staining. Microscope: Leica DM6000B, Wetzlar, Germany; Ex/Em: 358/460 nm; Objective: HC PL APO 63× 1.40 Oil, scale bar = 10 µm.

**Figure 5:**
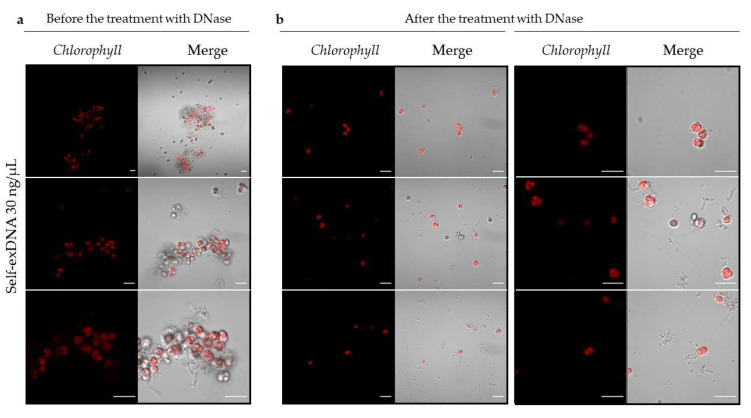
Microscopy analysis of *C. reinhardtii* aggregates after treatment with DNase. Aliquots of *C. reinhardtii* cells treated with self-exDNA at 30 ng/µL were observed before (**a**) and after the treatment (**b**) with 6 µL of 2.25 U/µL RNase-Free DNase (Qiagen) for 1 h. After treatments with DNase, cell aggregates break down. In red, the chlorophyll autofluorescence is shown (Ex/Em: 405/300–800). Microscope: Zeiss LSM 700, Oberkochen, Germany; excitation at 405 and detection in the range 300–800; objective: 63× 1.40 Oil Dic; scale bar = 10 µm.

For further analyses, we monitored the cell aggregate formation along time in the treatment of *C. reinhardtii.* An aliquot of the cellular suspension was analyzed under microscope starting from 48, to 96, and to 168 hpts by using DAPI staining and FITC filtering (Figure 4). The presence of palmelloid cells was evident already at 48 hpt, and, interestingly, the formation of aggregates surrounded by an extracellular matrix appeared since this stage. At 96 hpt, the cellular aggregates became more evident and the DAPI also diffusely stained the extracellular formations. At 168 hpt, the heteromorphous formations acquired a more evident and structurally defined organization with swelling cells with no evidence of nuclei (Figure 4, 168 hpt and Figure 5a). Interestingly, 1 hpt after the RNase-Free DNase treatment of aggregates formed at 168 hpt, in self-exDNA at 30 ng/µL, it resulted in disaggregation of the extracellular matrices embedding the *C. reinhardtii* cells (Figure 5b).

## 3. Discussion

### Inhibitory Effect of Self-exDNA in Microalgae

The exDNA has been found in nearly all terrestrial and aquatic habitats [2]. In marine and freshwater environments it is reported to be freely present in the water column as well as in sediments [62,63,66,67,68,70]. Its persistence in the environment is influenced by surrounding conditions, for example, the adsorption to particles [71,72], the presence of bacterial nucleases [75,76], and the temperature and salinity of the water [76,77]. Most of the recently published efforts describe the exDNA as a mineral source of phosphate [69] and carbon [81]. In the context of marine microbial communities or when located in the extracellular polymeric substance of marine biofilms, exDNA is also considered a source for genetic recombination and horizontal gene transfer [79,83].

Mazzoleni and colleagues provided the first demonstration showing that the exposure to fragmented self-exDNA triggers inhibitory effects on conspecifics, while the treatment with nonself-exDNA did not show similar effects [84,86]. The authors first described the inhibitory effect in plants [84] and subsequently confirmed similar effects also in species of different taxonomic groups, including the green alga *Scenedesmus*, suggesting a general biological phenomenon with ecological and evolutionary implications [85].

The exDNA has been demonstrated to be sensed in animals by receptors located in various cellular compartments, such as the nucleus [90,91], the cytoplasm [92,93,94], and the endosomes [95].

Nevertheless, in plants, no specific DNA receptor has been reported yet even if defensive pathogen-related (PR) proteins released by plants in the extracellular environment and plant pattern recognition receptors (PRRs) are considered good candidates as exDNA receptors [96].

Nevertheless, the possible mechanisms that allow cells or individual organisms to differently sense self- or nonself-exDNA, triggering a growth inhibition after exposure to self-exDNA, are still under investigation. Several hypotheses have been proposed to explain the early response to exDNA and the inhibitory effect of conspecific exDNA when compared with nonself-exDNA.

In bacteria, the perception and recognition of exogenous DNA is widely documented. In order to recognize foreign DNA, such as the viral genomes, bacteria are known to recognize differential patterns in the DNA structure. Usually unmethylated or differently methylated DNA of exogenous DNA are recognized through the DNA restriction–modification [97] and/or by the CRISPR–Cas systems [98,99,100]. *Cis* elements as the *Chi* sequences may be recognized by the RecBCD recombination system, and may characterize the bacterial DNA because of their higher frequency and their absence in phages [101], explaining specificity in the recognition. In addition to the above-mentioned systems, bacteria can also keep track of invasive elements by specific transcriptional silencing of horizontally acquired genes or prophages recombined with their own genome through the recognition of different compositional patterns, such as the higher A-T contents in foreign molecules, thus silencing them by the binding of repressor proteins (i.e., the heat-stable nucleoid-structuring protein [102]) or through the action of transcription termination factor (i.e., Rho protein [103]). Nevertheless, these process do not justify growth inhibition.

The sensing of exDNA molecules and the different processes triggered by self or nonself-exDNA, inhibition included, were suggested to be ascribed to mechanisms similar to sequence-specific recognition between small-sized nucleotide molecules, which could justify the specific inhibitory roles of extracellular self-exDNA in terms of the well-known processes of nucleic acid interference-like mechanisms [84,89,104], therefore justifying by a simple hybridization-like process the phylogenetic distance-related effects. Self-exDNA inhibition in plants was also suggested to be triggered by a costly immunological response [105,106].

Recently, the analysis of the molecular response of tomato [90] and *A. thaliana* [91] after treatments with self-exDNA added more molecular evidence to the current efforts in the field. In the first case, a similarity with biotic stress response was proposed.

In *A. thaliana* [89], the authors depicted complex and different cascades of events emerging from the molecular response to self- or nonself-exDNA. They observed different patterns of exDNA localization by fluorescence microphotography, with nonself-exDNA (non-similar or phylogenetically distant) entering root tissues and cells, while self-exDNA (conspecific and/or similar or “homologous”) remained outside. In addition, transcriptome analyses revealed that specific and different molecular pathways are triggered by the early response to self- and nonself-exDNA, respectively. They proposed to define these pathways with the new acronym EDAP, i.e., Extracellular DNA Associated Pathways, underlining that no specific pathways had been yet described that could explain the difference between the two categories of molecules (self and nonself-exDNA, respectively). Specifically, in the case of nonself-exDNA, the authors describe a remarkable differential gene expression, involving both biotic and abiotic stress-related genes, accompanied by the mounting of a hypersensitive response, putatively triggering a systemic acquired resistance. On the other hand, they demonstrated that self-exDNA triggers oxidative stress and the activation of the chloroplast genes, with the down-regulation of stress responsive genes [89], phenomena associated to Ca^2+^ spike signals and cell membrane depolarization at 30 min after exposure to self-exDNA, as demonstrated by other authors [87]. This is accompanied by the downregulation in signal transduction-related genes, suggesting decreased intracellular dynamics, possibly affecting the crosstalk between the chloroplast and the nuclear genome activities. They suggest that the lack of the up-regulation of genes acting as chloroplast ROS scavenging was mainly due to the inhibition of the chloroplast–nuclear cross-talk linked to O_2_ drop down, which may contribute to the activation of the NO pathway determining the downregulation of ethylene response after exposure to self-exDNA. The inhibition of the intracellular dynamics and crosstalk can justify the inhibitory effect of self-exDNA on cell growth and the extent of the damage to the whole organism. The process finds confirmation by the overproduction of chloroplast-related ROS, confirmed by overproduction of H_2_O_2_ reported in similar experiments in common bean [55] and in tomato [88], and can be associated with the lack of the overexpression of genes like ascorbate peroxidases (APX1) that could act as a scavengers of H_2_O_2_ in the chloroplast, as shown in *A. thaliana* [89], causing a not efficient ROS elimination and, ultimately, cell or DNA-damaging effects determining cell cycle arrest and growth inhibition observed at the macroscopic level [89]. Similarly, in tomato, the ubiquinol oxidase and catalase, which are major ROS scavengers in plants, were found downregulated in response to the treatment with self-exDNA [88], and this is probably directly correlated to the increased ROS production together with an impaired removal observed in tomato leaves. Interestingly, a very recent investigation highlighted that the treatment with self-exDNA in the model organism *Caenorhabditis elegans* affects reproductive behavior inducing cell damage and cell death [107], revealed by double-strand breaks demonstrated by the activation of RAD-51 and apoptotic nuclei.

As discussed in Chiusano et al., 2021, the different response to self- or nonself-exDNA exposure in *A. thaliana* appears to start in the extracellular environment, possibly on the extracellular surface of the plasma membrane or on its immediate surroundings, explaining the reasons for the accumulation of extracellular self-exDNA on cell surfaces, in contrast with the entrance and the active endocytosis stimulated by the nonself-exDNA. This justifies different molecular mechanisms associated with the different molecular responses. On this basis, the authors also speculated that nucleic acids known to be present in the extracellular environment could be directly involved in the self/nonself-exDNA sensing in plants, and presumably in all living beings [21].

However, further validation on the location and the nature of the possible receptors involved in specific sensing of exDNA is needed.

The first insight of the inhibitory effects of self-exDNA in aquatic environments was highlighted by Mazzoleni and his colleagues in 2015 [86] and also on the green alga *Scenedesmus* growth, in comparison with the exposure to nonself-exDNA, which did not exert any evident effect on the algal growth. Nevertheless, beyond this first confirmation, the analysis of the microalgae growth over time (i.e., through the evaluation of growth curves) as well as the microscopic evaluation of any cellular morphological change and the confirmation of an inhibitory effect in both freshwater and marine environments following the treatment with self-exDNA in comparison with nonself-exDNA was never explored up to now.

Our results showed that the presence of self-exDNA in mineral growth medium affects cell growth in both freshwater and marine microalgae. Indeed, cell growth was reduced and the generation time increased. Indeed, the growth inhibition rate was almost the 80% for both microalgae species when compared with the control, and the generation time increased by almost 5 times more than in the control at the highest concentrations of DNA (30 ng/µL). Interestingly, this inhibition appeared to have a concentration-dependent effect in *N. gaditana*. In *C*. *reinhardtii*, the self-exDNA at 10 ng/µL exerted the highest inhibitory effect at 96 hpt, while, at the same concentration, the cell growth overcame the control at 168 hpt when cell density was higher than the control, demonstrating a possible recovery from the inhibitory effect. The cell growth seemed to be favored also in the presence of very low concentrations of self-exDNA (3 ng/µL) in the medium. This could be interpreted as a prevalence of a nutritive advantage at very low concentration that opposes the evident inhibitory effects at higher concentrations. These results, while confirming the remarkable inhibitory effect in both species at higher self-exDNA concentrations, also revealed the positive effects of lower concentrations due to the well-known nutritive role of exDNA, as extensively discussed in the literature [2,10,69]. This can also explain the positive recovery from the self-exDNA inhibition revealed in *N. gaditana* at 10 ng/µL considering 96 and 168 hpts.

It is worth noting that the cell growth at 168 hpt in all the tested concentrations of nonself-exDNA seemed to slightly promote *C*. *reinhardtii* cell growth, while reducing the growth in *N. gaditana.* This highlights that species can have different sensitivity to DNA exposure and that the nature of the DNA, as well as the environmental context in which this exposure takes place, can affect species growth and generation time, paving the way for the need for further investigations to highlight the possible different effects due to molecular phylogenetic distances and environmental physico-chemical conditions.

Interestingly, microscopy analyses revealed the formation of heteromorphous organizations after treatments with self-exDNA at 30 ng/µL. These organizations appear to be formed by both morphological altered cells and extracellular matrices forming aggregates in both species. Interestingly, we highlighted the presence of palmelloid cells within the aggregates formed when treating *C*. *reinhardtii*. It is well-known that *C*. *reinhardtii* cells show palmelloids phenotypes in multicellular aggregates when exposed to stress agents, such as organic acids [108], chloroplatinic acid [109], herbicide paraquat [110], and in salt stress [111], as well as in phosphate-limited growth medium [112]. These cells, while proceeding through cell division, appear to be unable to complete their separation, thus remaining enclosed within a common cell wall [111]. Accordingly, the treatment with self-exDNA at 30 ng/µL seems to determine a similar cell phenotype, putatively arresting cell growth and determining an uncomplete development. Nevertheless, other molecular and morphological analyses are required to better characterize the formation of palmelloids in *C*. *reinhardtii*, for example, investigating to what extent the cell cycle progression is affected by the exposure to higher concentrations of self-exDNA.

Concerning the extracellular aggregates formed only after treatment with self-exDNA at 30 ng/µL, our temporal analysis revealed that palmelloids cells and the extracellular matrix started to form at 48 hpt, becoming progressively evident and clearly defined up to 168 hpt, where swelling cells without nuclei appeared. The presence of exDNA within these aggregates was confirmed by both DAPI staining and by their break down after the treatment with DNase. For these structure features, these heteromorphous aggregates resemble extracellular matrices recalling microbial biofilms of both prokaryotes [35,113] and eukaryotes origin [114], as well as RETs, i.e., root extracellular traps, in plants [14], and NETs, i.e., neutrophil extracellular traps, in mammals [3], where it is widely reported that the exDNA has both a structural and a functional role [27].

On the basis of the observations of the inhibitory effects of self-exDNA [84,86], and of our results reporting that aggregates of cells and extracellular matrices organize after exposure to self-exDNA, and that DNA is essential for maintaining these formations, we hypothesize that self-exDNA-mediated growth inhibition may facilitate the formation of aggregates. These aggregates are possibly organized by extruded material, DNA included, forming extracellular structures that break down thanks to nucleases, as also reported in other efforts [4,115,116], confirming the crucial role of DNA molecules in favoring the formation of these structures. It was also proposed [27] that these matrices, favoring the trap of free self-exDNA, limit its bio-availability within the environment as a free molecule and, therefore, its effect as growth inhibitor [27], favoring the hypothesis that biofilm formation could represent an evolutionary outcome providing and advantage in adverse conditions. In addition, this also confers a protective role, since the exDNA in these matrices has been shown to exert a protective role, impeding pathogen attacks [4,29]. Indeed in plants it was demonstrated that, upon DNase treatments, the degradation of the DNA in the RETs matrices results in the loss of root tip resistance, thus favoring pathogens infection [4]. Additionally, the removal of DNA in NETs matrices decreases their formation and downstream favorable effects (i.e., the activation of the complement system) [117]. Moreover, it has been demonstrated that treatments of NETs with an excess of cations or phosphatase enzyme, and exogenous or secreted microbial DNases, protects pathogens from the NET antibacterial action [118]. These independently described pieces of evidence highlight that all these phenomena may be deeply crosslinked and should be considered as an overall picture, suggesting that these processes could have evolved because they limit the inhibitory effects of the presence of self-exDNA in the environment, meanwhile favoring protection of the individuals.

The formation of these aggregates, also composed of exDNA, and appearing after the treatment with self-exDNA at 30 ng/µL, show the main effects of a mechanism involving a specific recognition of the nature of the exDNA (i.e., conspecific or heterospecific), also recently discussed in higher plants [89]. Further molecular and morphological analyses should be performed to better characterize *C. reinhardtii* cells within aggregates (i.e., palmelloids cells) as well as the aggregates composition in the two species. Moreover, future investigations are still required to clarify the mechanism of differential perception involved in the sensing of self- and nonself-exDNA, the confirmation of similar effects with other nonself nucleic acids, and a better characterization of the nature of these aggregates and the molecular processes involving their formation.

## 4. Impact and Future Perspectives

Considering the body of information related to multicellular organisms exposure to self and nonself-exDNA, and the similar inhibitory effects revealed in unicellular species (to *Trichoderma harzianum* and *Scenedesmus* [86]), the analysis here presented for two microalgae growing at different salt concentration confirms the universality of the process involved in the responses, offering suitable photosynthetic model systems to deeper understand the possible molecular mechanisms involved in extracellular sensing and ROS formation. In addition, as hypothesized in Monticolo et al., 2020 [27], the presence of self-exDNA could trigger the formation of cell aggregates mediated by etheromorphous cell-secreted substances that could restrict the extent of inhibition exerted by self-exDNA to exposed cells.

Furthermore, from previous observations on the relevance of the phylogenetic distance in influencing the inhibitory effect, as also reported in the introduction, we could assume that the DNA from other microalgae would have an inhibitory effect on either *C. reinhardtii* or *N. gaditana* and that the degree of the inhibition would be in accordance with the phylogenetic distance from the target species. These observations, together with the need of a deeper view on the mechanism through which the self- and nonself-exDNA act, raise the interest for further investigations, both at transcriptomic and microscopic levels, of microalgae response after the exposure to nonself-exDNA at different degrees of phylogenetic distance. For these reasons, our results showing the inhibitory effect of self-exDNA in aquatic species also have great ecological and biotechnological implications. Indeed, the evidence of the inhibitory effect of self-exDNA paves the way to novel investigations on ecological issues impacting ecosystems biodiversity and biocontrol in aquatic environments. In addition, the development of strategies focusing on removal of self-exDNA to avoid growth inhibition or biofilm formation may be useful in industrial applications.

## 5. Materials and Methods

### 5.1. DNA Extraction and Fragmentation

The genomic DNA from *C. reinhardtii* and *N. gatitana* (self-DNA) was extracted adapting the protocols of Jagielski, 2017 [119] and Jeffrey Doyle, 1991 [120]. The algal cells were collected and ground with pestle in a pre-chilled mortar in presence of Polyvinylpyrrolidone (PVP) powder and re-suspended in pre-warmed CTAB isolation buffer (2% CTAB, 1.4 M NaCl, 100 mM Tris pH 8.0, 20 mM EDTA) for 1 h in a water bath set at 60 °C. The DNA was extracted within an equal volume of chloroform-isoamyl alcohol (24:1) and centrifuged 15 min at max speed. The supernatant was collected and the DNA was precipitated overnight at −20 °C with two volumes of 100% ethanol *v*/*v* and 1/10 of 3 M NaOAc pH 5.2. The ethanol was removed after 15 min of centrifugation at max speed (4 °C), and the pellet was washed with 70% ethanol *v*/*v* and centrifuged for 10 min at max speed. The pellet was air-dried and then dissolved in sterile MilliQ water. The nonself-exDNA was extracted from the *Sardina pilchardus* (nonself-DNA) abdominal muscle following the same procedure used for microalgae.

After DNA extraction, the re-suspended DNA was treated with RNase A (Thermo Fisher) for 1 h at the final concentration of 0.25 mg/mL at 37 °C.

The DNA was extracted with the same procedure from different tissue types to randomize and overcome biases due to the extraction procedure itself. From 8 to 10 independent DNA extractions were performed per sample type (technical replicates). DNA purity was assessed with nanodrop standard quality parameters (260/280 and 260/230 ratios all always above 1.8 and 1.7, respectively). The DNA quantity was evaluated using a QUBIT (Thermo Fisher) fluorimeter, and its integrity was evaluated by electrophoresis in 1% (*w*/*v*) agarose gel (Supplementary Figure S2). The DNA was sheared using a Bioruptor Plus (Diagenode), in 12 min at the high power setting, 60 sec ON and 30 sec OFF [84].

### 5.2. Algal Growth and Growth Inhibition Assay

Axenic cultures of *Chlamydomonas reinhardtii* (strain CCAP 11/32b) and *Nannochloropsis gaditana* (strain 639) obtained from the Algal Collection University Federico II were grown in Bold’s Basal Medium (BBM) and Artificial Seawater Medium (ASW) both supplemented with vitamins, respectively at 24 ± 1 °C, on a rotary shaker at 100 rpm with a photoperiod of 16:8 h light/dark at 100 E m^−2^ s^−1^; subculturing was maintained weekly in fresh medium [121,122].

**Figure 6:**
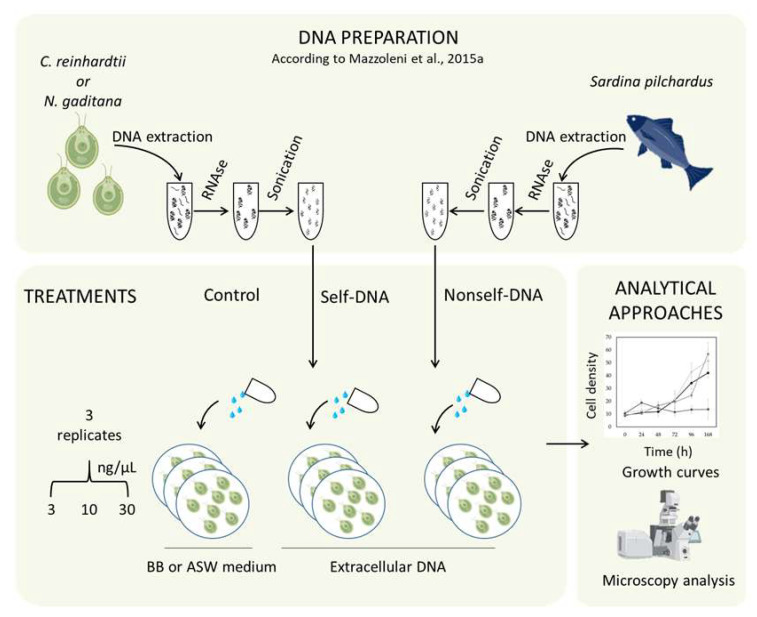
Schematic overview of the experimental design. DNA PREPARATION: extraction, RNase treatment, and sonication of DNA from *C. reinhardtii* or *N. gaditana* (self-DNA) and from *Sardina pilchardus* (nonself-DNA), according to Mazzoleni et al., 2015a [86]. TREATMENTS: the exposure of both microalgae to three different concentrations (3, 10, and 30 ng/µL) was performed in triplicate. ANALYTICAL APPROACHES: Growth curves of microalgae and microscopy analyses were performed to evaluate the effects of both exDNA. Legend: BB medium is Bold-Basal medium and ASW is Artificial Seawater Medium.

Growth inhibition assays were performed based on the modified protocol reported in Nunes et al. in 2014 [123]. Specifically, the assay was performed in 24-well microplates and, for each treatment, three replicates were assayed; then, plates were incubated for 168 h. The cell density was estimated by an indirect method, i.e., measuring the optical density using a Multiskan Sky Microplate Spectrophotometer (Thermo Scientific^TM^, Waltham, MA, USA) every 24 h by taking an aliquot from each treatment that was fine resuspended by pipetting and transferred in a 96 flat-bottomed well, used for light absorbance measurements. Each microalgae species was treated with fragmented self and nonself-ex DNA at 0, 3, 10, and 30 ng/µL in triplicates. A schematic overview of the experimental design is represented in Figure 6.

The specific growth rate (µ) was calculated for each species according to the following equation:

$$\mu =\frac{\ln N_{2}-\ln N_{1}}{t_{2}-t_{1}}$$

where *N*_2_ and *N*_1_ represent the cell density (cell ml^−1^) at time *t*_2_ and *t*_1_ (expressed in hours).

The generation time (Tg) was calculated based on the following equation:

$$\mathrm{Tg}=\frac{\ln2}{µ}$$

The percentage of inhibition (%I) for both species was calculated with respect to control at 96 h post treatment (hpt) and at 168 hpt, using the following equation:

$$\%\;I=\left[\frac{\left(\mu_{c}-\mu_{t}\right)}{\mu_{c}}\right]*100$$

with $\mu_{c}$ representing the specific growth rate in the control group and $\mu_{t}$ the specific growth rate in the DNA treatment, respectively.

Statistical analyses were performed by Shapiro–Wilk and Bartlett test using the SPSS 27.0 software.

### 5.3. Microscopic Analysis of Cell Structure and Chlorophyll Distribution

Morphological analyses were performed with two independent microscopes by using a 63× oil immersion objective. The confocal microscope Zeiss LSM 700, Oberkochen, Germany, was used to observe the cellular chlorophyll distribution (Ex/Em: 405/300–800 nm) and the microscope Leica DM6000B, Wetzlar, Germany was used to observe cells using FITC and DAPI filters (Ex/Em: 488/525 nm and 358/460 nm, respectively)

### 5.4. DNase Treatment

*C. reinhardtii* cell aggregates were analyzed at 168 hpt with self-exDNA at 30 ng/µL. 13.5 U of RNase-Free DNase (Qiagen, Hilden, Germany) were added to the microalgae cell culture and a phenotypic analyses were carried out using the microscope Leica DM6000B, Wetzlar, Germany, 1 h post DNase treatment. All analyses were performed at least in triplicates to confirm the results.

## Figures and Tables

**Table 1:** Microalgae growth parameters (index). Average cell density (×10^4^ cells mL^−1^), generation time (Tg), and growth rate inhibition measured as the % with respect to the control (GRI (%)) of *C. reinhardtii* and *N. gaditana* at 168 h post treatment (hpt), at different concentrations of nonself- and self-exDNA; sd: standard deviation. Different letters indicate statistically significant differences between treatments in each column.

	*C. reinhardtii*			*N*. *gaditana*		
Treatment (exDNA ng/µL)	Average Cell Density (×10^4^ Cells mL^−1^ ± sd)	Tg (Days ± sd)	GRI (% of Control ± sd)	Average Cell Density (×10^4^ Cells mL^−1^ ± sd)	Tg (Days ± sd)	GRI (% of Control ± sd)
Contr	42.2 ± 3.6 ^b^	3.6 ± 0.2 ^b^	-	65.3 ± 9.8 ^a^	6.7 ± 0.2 ^e^	-
Nonself 3	42.7 ± 3.3 ^b^	3.8 ± 0.4 ^b^	6.3 ± 9.2 ^a^	52.8 ± 9.8 ^c^	10.2 ± 0.2 ^c^	11.0 ± 1.5 ^b^
Nonself 10	45.7 ± 2.8 ^b^	3.4 ± 0.1 ^b^	−4.8 ± 3.8 ^a^	57.6 ± 9.8 ^b^	8.8 ± 0.3 ^d^	28.0 ± 2.8 ^a^
Nonself 30	52.9 ± 7.7 ^a^	3.2 ± 0.4 ^b^	−13.1 ± 12.3 ^a^	59.7 ± 19.6 ^b^	8.5 ± 1.3 ^d^	14.0 ± 10.2 ^a^
Self 3	51.8 ± 8.6 ^a^	3.6 ± 0.7 ^b^	−1.6 ± 19.2 ^a^	58.0 ± 13.0 ^d^	10.3 ± 0.3 ^c^	2.0 ± 1.7 ^c^
Self 10	57.1 ± 8.7 ^a^	3.0 ± 0.3 ^b^	−17.8 ± 9.7 ^a^	51.2 ± 11.0 ^c,e^	11.7 ± 0.3 ^b^	28.0 ± 1.6 ^a^
Self 30	13.7 ± 0.6 ^c^	22.4 ± 3.3 ^a^	83.7 ± 2.7 ^b^	42.2 ± 13.2 ^f^	36.6 ± 11.2 ^a^	81.6 ± 9 ^d^

## Data Availability

Data sharing not applicable to this article as no datasets were generated or analyzed during the current study.

## Supplementary Materials

The following are available online at https://www.mdpi.com/article/10.3390/plants11111436/s1. Figure S1. Microscopy analysis of *C. reinhardtii* (a) and *N. gaditana* (b) samples in controls and after treatment with nonself- and self-exDNA at 3, 10 and 30 ng/µL. In red, the chlorophyll autofluorescence (Ex/Em: 405/300-800). Microscope: Zeiss LSM 700, Oberkochen, Germany, Excitation at 405, detection in the range 300-800, scale bar = 10 µm, Objective: 63× 1.40 Oil Dic. Figure S2. Agarose gel electrophoresis of the DNA extracted from *C. reinhardtii* and *N. gaditana* cells. Figure S3. Fluorescence analysis of *C. reinhardtii* (a) and *N. gaditana* (b) samples in the controls and after treatments with self-exDNA at 30 ng/µL. Controls are represented by microalgae grown in BB and ASW media for *C. reinhardtii* (a,b) and *N. gaditana* (c,d), respectively Cellular aggregates were analysed using DAPI staining. Microscope: Leica DM6000B, Wetzlar, Germany, Ex/Em: 358/460 nm, Objective: HC PL APO 63× 1.40 Oil, scale bar = 10 µm.
