# Supplementary figures and images for: Effects of Extracellular Self- and Nonself-DNA on the Freshwater Microalga Chlamydomonas reinhardtii and on the Marine Microalga Nannochloropsis gaditana
Source: Plants (Basel). 2022 May 27;11(11):1436. doi: 10.3390/plants11111436 (PMC9183124; doi:10.3390/plants11111436)

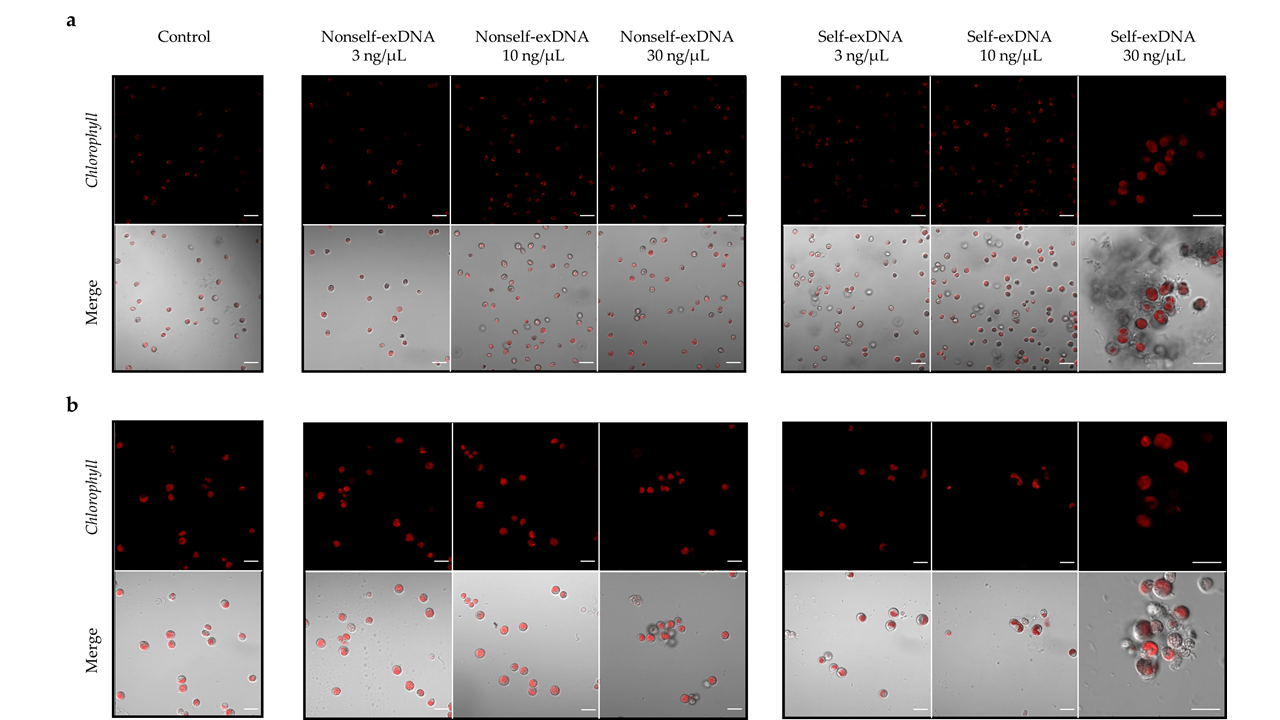

Supplement: Supplementary file 1 [file plants-11-01436-s001.zip › Supplementary Figure S1.tif]

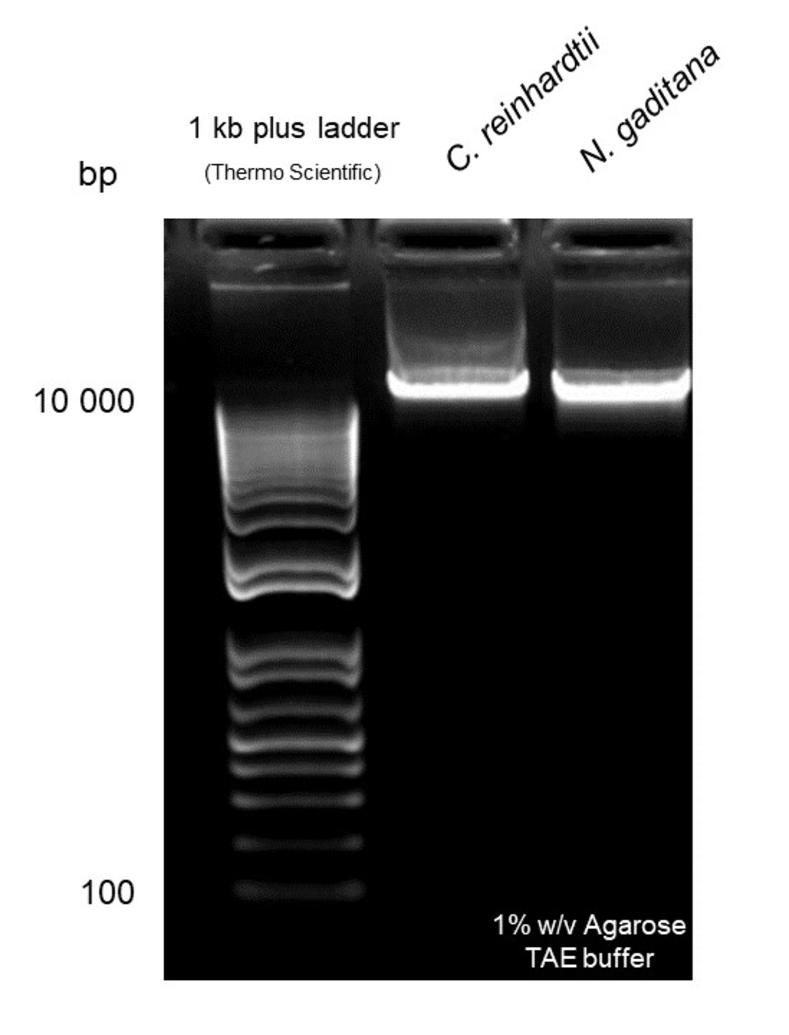

Supplement: Supplementary file 1 [file plants-11-01436-s001.zip › Supplementary Figure S2.tif]

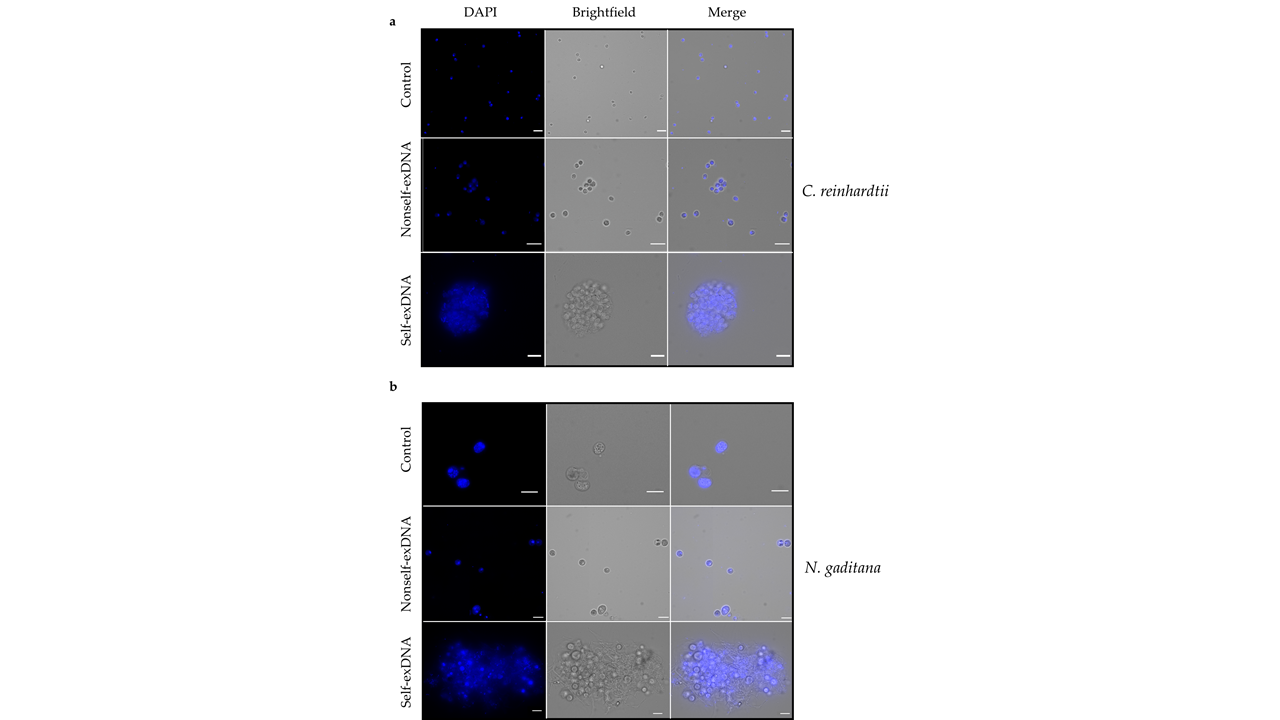

Supplement: Supplementary file 1 [file plants-11-01436-s001.zip › Supplementary Figure S3.tif]
